# Exacerbations and Lung Function in Polish Patients with Chronic Obstructive Pulmonary Disease Treated with ICS/LABA: 2-Year Prospective, Observational Study

**DOI:** 10.3390/jcm14238544

**Published:** 2025-12-02

**Authors:** Piotr W. Boros, Rafał Pawliczak, Tomasz Dębowski

**Affiliations:** 1 Lung Pathophysiology Department, Institute of Tuberculosis and Lung Diseases, Plocka 26, 01-138 Warsaw, Poland; 2 Department of Immunopathology, Faculty of Medicine, Division of Biomedical Science, Medical University of Lodz, 7/9 Zeligowskiego St., 90-752 Lodz, Poland; rafal.pawliczak@csk.umed.lodz.pl; 3 Chiesi Poland sp. z o.o., Aleje Jerozolimskie 134, 02-305 Warsaw, Poland; t.debowski@chiesi.com

**Keywords:** chronic obstructive pulmonary disease, COPD, ICS/LABA, exacerbation, GOLD, spirometry, ATS/ERS

## Abstract

**Background/Objectives:** To assess the frequency of exacerbations of chronic obstructive pulmonary disease (COPD) and their association with lung function in patients treated with ICS/LABA in everyday medical practice in Poland, we conducted a prospective observational study. **Methods:** Patients diagnosed with COPD for at least 12 months before enrolment and ambulatory treatment with ICS/LABA for at least 6 months before study entry were followed up for 2 years after the initial visit. At four subsequent visits, data on pulmonary function, exacerbations, and symptoms were collected. The severity of airflow limitation was assessed using the %pred. (GOLD) and z-scores (ATS/ERS 2022). **Results:** At each visit, approximately 80% of the patients had an mMRC ≥ 2 and CAT score ≥ 10. In 330 patients defined as ‘decliners’, a decrease in FEV1 greater than 100 mL was observed. At the initial visit, 76.5% of patients reported exacerbation of COPD in the 12-month period preceding study entry. At each subsequent visit, fewer exacerbating patients reported hospitalization (from 27.7% at Visit 2 to 18.4% at Visit 4). Regression analysis revealed that the presence of comorbidities and higher mMRC values (OR = 1.556 [CI:1.099–2.203], *p* = 0.013 and OR = 2.656 [CI:2.163; 3.262], *p* < 0.001, respectively) were independent factors associated with COPD exacerbations. **Conclusions:** During the 2-year period, pulmonary function and patient-related outcomes, such as severity of dyspnea measured by mMRC score and CAT, were generally stable throughout the study. Symptoms and comorbidities, but not lung function, were associated with the risk of exacerbation.

## 1. Introduction

Chronic obstructive pulmonary disease (COPD) is a respiratory disorder characterized by irreversible and progressive airflow limitation [1]. COPD is the most common chronic respiratory disease in older adults and the third leading cause of death worldwide [2,3]. Patients with COPD commonly present with dyspnea, chronic cough, and sputum production; their lung function declines, which is routinely assessed by spirometry. Although COPD has different clinical forms and levels of airway obstruction severity, many patients experience exacerbations that lead to hospitalization [4], and the disease significantly decreases the health status of patients [5]. According to the GOLD 2025 definition, COPD is a heterogeneous, preventable, and treatable disease characterized by persistent respiratory symptoms and airflow limitation. Acute exacerbations (AECOPD) are now recognized as the main drivers of lung function decline and disease progression and are responsible for up to 60–70% of total COPD-related healthcare expenditures and a substantial proportion of hospitalizations and mortality worldwide [6]. The current recommendations of the American Thoracic Society/European Respiratory Society (ATS/ESR) present a new system for the evaluation of lung function impairment severity that does not use the percentage of predicted FEV1 values but focuses on z-score values [7].

The Global Initiative for Chronic Obstructive Lung Disease (GOLD) recommends maintenance therapy with different types of bronchodilators depending on the disease severity phenotype (short-acting bronchodilators, long-acting beta agonists [LABAs], or long-acting muscarinic antagonists [LAMAs]). For patients with repeated exacerbations, combination treatment with inhaled corticosteroids (ICS) such as ICS/LABA or ICS/LABA/LAMA should be considered [4].

The efficacy of ICS/LABA in COPD treatment has been widely studied for many years. It has been shown that these drugs’ combination reduces the exacerbation rate and improves the health status and spirometric characteristics of lung function of COPD patients [8,9,10,11,12]. However, there are a small number of real-life longitudinal (>12 months) observational studies aimed at evaluating the effectiveness of ICS/LABAs in COPD populations. In particular, there is a lack of studies on Polish COPD patients [13]. Moreover, clinical studies applying new ATS/ERS recommendations for the evaluation of lung function impairment severity have not yet been conducted.

The purpose of this observational, non-interventional study was to assess the frequency of COPD exacerbations and hospitalizations due to these exacerbations and to evaluate changes in lung function for a 2-year observation period in patients treated with ICS/LABA in everyday medical practice in Poland. Additionally, the risk of COPD exacerbations was assessed using the new strategy of pulmonary functional test interpretation recommended by the ATS/ERS in 2022.

## 2. Materials and Methods

### 2.1. Study Design and Participants

This open, prospective, multicenter, non-interventional observational study enrolled 965 patients who were diagnosed with COPD for at least 12 months before enrolment and were treated with ICS/LABA for at least 6 months prior to enrolment. The study lasted 24 months (2015–2017), during which patients had five visits (visits 0–4) to the treating physician.

The study was performed in accordance with the Declaration of Helsinki, the International Conference on Harmonization Harmonized Tripartite Guideline for Good Clinical Practice, and local regulations. This is a prospective observational, non-interventional, real-life study, so, according to Polish law, the Local Ethical Committee does not have to approve the study protocol. Additionally, in this type of study, the informed consent of the participants was not required at the time of conducting the observation.

Patients were excluded from this study if any of the following criteria were present: (i) had bronchial asthma, (ii) had a contraindication to treatment with an ICS/LABA combination, (iii) were pregnant or lactating, and (iv) were currently participating in another clinical trial. If any of the abovementioned criteria were met, the patient was excluded from the study.

### 2.2. Endpoints and Measurements of the Study

**Figure 1:**
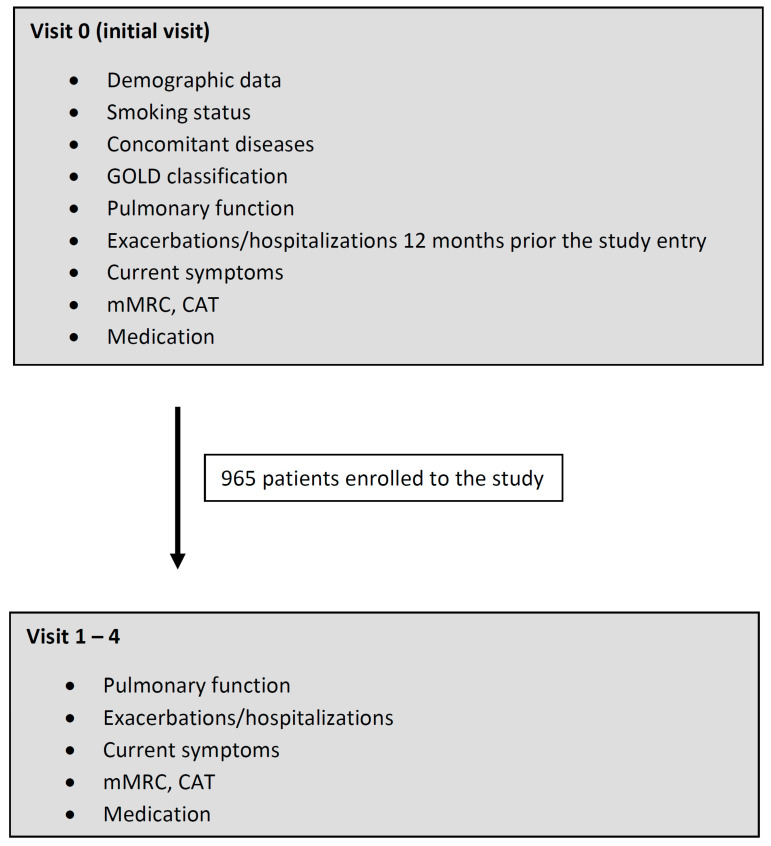
Data collected during initial visit and next visits.

Patients were enrolled in the study at visit 0 (the initial visit). Six months after the initial visit, visit 1 was scheduled. Subsequent visits (Visit 2–4) were performed every 6 months (+/− 14 days). Patient data collected during the initial and subsequent visits are presented in the flow chart (Figure 1).

The primary endpoints of the study were a change in pulmonary function parameter FEV1 and the frequency of COPD exacerbations and hospitalizations due to exacerbations. Secondary endpoints included assessment of GOLD category distribution, type of comorbidities, and degree of stability of COPD treatments. The study variables were as follows: FEV1 and FVC (expressed in absolute values, % predicted and z-scores) applying Global Lung Function Initiative (GLI) equations [14], number of exacerbations and hospitalizations, demographic data of patients and data on the following: (i) smoking habits, (ii) concomitant diseases, (iii) symptoms currently occurring, (iv) level of dyspnea’s severity assessed by modified Medical Research Council (mMRC) scale, (v) COPD Assessment Test (CAT) score, (vi) current treatment, (vii) treatment with antidepressants, (viii) classification to GOLD 2017–2022 groups, and (ix) adverse drug reactions.

In this study, it was assumed that the FEV1/FVC ratio in obstructive patients should be ≤0.7. Additionally, patients were categorized as ‘decliner’ or ‘non-decliner’ depending on the occurrence of FEV1 loss greater than 100 mL during the study. Based on GOLD and new ATS/ERS recommendations [6], patients were divided into four categories based on spirometry values:

- non-severe; ATS/ERS-non-severe (FEV1 > 50% pred. and z-score > −4);
- GOLD-non-severe: ATS/ERS-severe (FEV1 > 50% pred. and z-score < −4);
- GOLD-severe—ATS/ERS-non-severe (FEV1 < 50% pred. and z-score > −4);
- GOLD-severe: ATS/ERS-severe (FEV1 < 50% pred. and z-score < −4).

### 2.3. Statistical Analysis

Descriptive statistics were provided for all the variables. Multivariate logistic regression was used to assess predictors of COPD exacerbation. The results of multivariate regression analysis are presented as odds ratios with respective 95% confidence intervals (CI). Poisson regression analysis was used to assess the predictors of pulmonary function. The results of the Poisson regression are presented as β coefficients and respective *p*-values. The results were considered statistically significant at *p* < 0.05. Given the observational RWE design and incomplete follow-up, missing data were handled according to available-case analysis; results should be interpreted with caution regarding potential selection bias. All calculations and analyses were performed using R 3.5 statistical software and MedCalc^®^ Statistical Software version 20.218 (MedCalc Software Ltd., Ostend, Belgium; 2023).

## 3. Results

### 3.1. Patients’ Characteristics and Progress of the Study

In total, 965 patients were enrolled in the study during their initial visit (visit 0). The majority of patients were males (67.7%), and the mean age of participants was 66.9 (9.4) years. Almost all patients (91.6%) were current smokers or ex-smokers. Among concomitant diseases, the most frequent were cardiovascular diseases, such as hypertension (70%), ischemic heart disease (30%), and heart failure (20%). Depression and anxiety disorders were diagnosed in 6.8% of patients.

Regarding the functional criteria, only 790 participants met the post-bronchodilator FEV_1_/FVC ≤ 0.70 threshold. The remaining individuals were included based on a documented clinical diagnosis of COPD and ongoing pharmacological treatment consistent with COPD management pathways. This reflects the well-described phenomenon of COPD overdiagnosis and treatment initiation without spirometric confirmation, which has been highlighted in real-world cohorts and observational studies [15,16,17].

Regarding medication used during the study, the combinations of ICS/LABA taken by study patients did not differ considerably from visit to visit and was noted in 96% to 100% of visit-to-visits. Extrafine beclomethasone/formoterol was used in up to 40% of study patients, and fluticasone propionate/salmeterol in about 20%. LAMAs were the most frequently used drugs as concomitant therapy (between 58.9 and 68.9% of patients from visit to visit), then short-acting beta2-adrenergic agonists (between 37.8% and 46.4% of patients) and aminophylline (between 35.5% and 45.7%). More details on the demographic characteristics, smoking status, concomitant diseases, and treatment methods at each visit are given in Table 1 and Table 2.

The number of patients decreased gradually from visit to visit. Eight hundred and sixty-seven (89.8%) patients were present at Visit 1, 707 at Visit 2 (73.3%), 536 (55.5%) at Visit 3 and 151 (15.6%) at Visit 4.

### 3.2. Exacerbations During the Study

**Figure 2:**
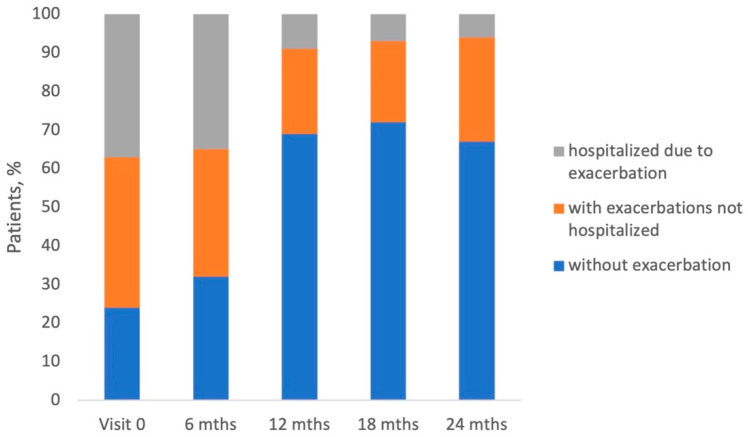
Proportions of COPD patients reporting no exacerbations (blue bar), exacerbations without hospitalization (orange bars), and severe exacerbations requiring hospitalization (gray bars). Proportions are demonstrated for each visit and are derived from the total number of 932 patients for Visit 0: 703 for 6-month, 578 for 12-month, 531 for 18-month, and 149 for 24-month visit.

At the initial visit, 76.5% of patients reported exacerbation of COPD in the 12-month period preceding the study entry. In the 6-month period between Visit 0 and Visit 1, this proportion was 68.3%, and in the 6-month period between all other visits—about 30%. About half of the patients experiencing exacerbations were hospitalized in the 12-month period preceding study entry and in the 6-month period between Visits 0 and 1. At each subsequent visit, fewer exacerbating patients reported hospitalizations (from 27.7% at Visit 2 to 18.4% at Visit 4) (Figure 2).

Patients with exacerbations in their history had more frequently comorbidities (81.4% vs. 67.9%, *p* < 0.001) and, less frequently, were active smokers (32.3% vs. 40.0%, *p* = 0.016). Multivariate regression analysis revealed that independent predictors of being classified into the C/D GOLD category (factors associated with the occurrence of exacerbations) were the presence of comorbidity and higher mMRC values (OR = 1.556 [CI:1.099; 2.203], *p* = 0.013 and OR = 2.656 [CI:2.163; 3.262], *p* < 0.001, respectively). Other factors taken into account, such as sex, age, BMI, severity of airway obstruction, the fact of smoking, and a significant decrease in FEV1, were found to be not significant in this model. Detailed data for multivariate analysis are provided in Table 3.

### 3.3. Pulmonary Function

At the initial visit, the value of FEV1/FVC ratio was equal or less than 0.7 in 790 patients (81.9%). This group of patients was included in further analyses of pulmonary function. Based on the FEV1 parameters, the proportions of patients with mild, moderate, severe, and very severe airflow limitation were comparable between visits (see Table 4).

In 330 patients, a decrease in FEV1 greater than 100 mL was observed, and these patients were defined as ‘decliners’. The comparison of ‘decliners’ with ‘non-decliners’ is presented in Table 5. ‘Decliners’ had significantly better lung function (FEV1 and FVC) than those with stable lung function; however, there were no differences in terms of exacerbations.

**Figure 3:**
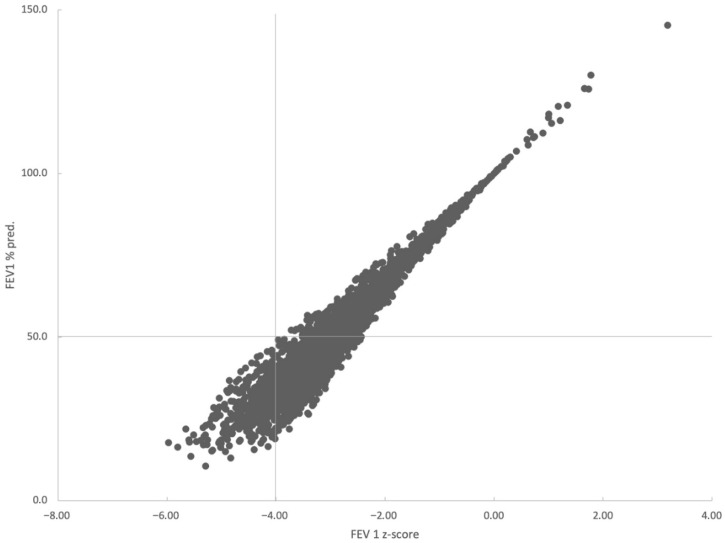
The distribution of patients categorized by two systems of lung function impairment severity: GOLD, based on FEV 1% of predicted value, and ATS/ERS based on FEV1 z-score. The horizontal and vertical lines indicate the threshold values for severe impairment according to ATS/ERS 2005 (50% of predicted) and ATS/ERS 2022 (z-score = −4), respectively.

The distribution of patients categorized by two systems of lung function impairment severity (GOLD and ATS/ERS) is presented in Figure 3. There were no patients categorized as GOLD-non-severe/ATS/ERS-severe (FEV1 > 50% pred. and z-score < −4). The comparison of patients’ GOLD/ATS/ERS categories is presented in Table 6.

### 3.4. COPD Symptoms, Severity of Dyspnea, and GOLD Classification of the Study Patients

In 36.4% of patients, the COPD symptoms occurred between 5 and 10 years before enrolment in the study. In approximately one quarter of the cases, the symptoms persisted between 2 and 5 years (25.7%) and between 10 and 20 years (25.6%). The mean time since the diagnosis was 6.42 (5.32) years.

The most common symptom of COPD at each visit was shortness of breath during exercise (approximately 90% of patients). According to GOLD 2017–2022 recommendations, at initial visit, the majority of the patients were categorized into the GOLD D group (49.8%), followed by GOLD B (41.2%), GOLD A (7.3%), and GOLD C groups (1.6%)—see Table 7).

**Figure 4:**
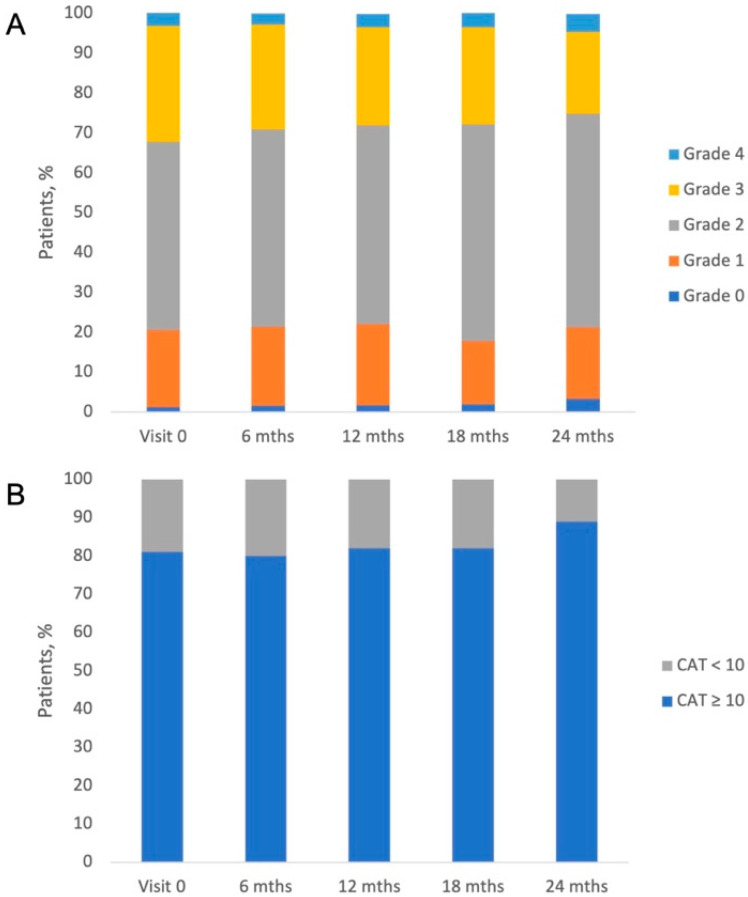
Percentages of patients declaring dyspnoe by mMRC level (**A**) and CAT scores (**B**) at subsequent visits.

The severity of dyspnea was assessed using an mMRC questionnaire and was stable from visit to visit. At each visit, about 80% of patients had an mMRC ≥ 2. About half of patients had mMRC grade 2. The second-most numerous groups were patients with mMRC grade 3 (about 30%), then with grade 1 (about 20%). The fewest number of patients had grade 4 (about 3%) or 0 (about 1.5%) dyspnea severity. In the CAT, patients obtained median scores from 15 to 17 points and these results were comparable for each visit (17 [IQR:11–22] for initial visit; 16 [11,12,13,14,15,16,17,18,19,20,21] at 6-month; 16 [11,12,13,14,15,16,17,18,19,20,21] at 12-month; 15 [10,11,12,13,14,15,16,17,18,19,20] at 18-month and 17 [13,14,15,16,17,18,19,20,21] at 24-month visit]). At each visit, at least 80% of patients had a CAT score ≥ 10. Figure 4 presents the proportions of patients with mMRC levels (A) and CAT more or less than 10 points (B).

## 4. Discussion

This non-interventional observational study aimed to assess the frequency of COPD exacerbations and hospitalizations, due to these exacerbations, and to evaluate the changes in lung function for the 2-year observation period in patients treated with ICS/LABA in everyday medical practice in Poland. Patient-related outcomes, such as symptoms, smoking habits, and type of treatment, were also recorded during this study. Moreover, in this publication, the risk of COPD exacerbations was assessed using a new strategy of pulmonary functional test interpretation recommended by the ATS/ERS in 2022 [7].

The results of our study demonstrated that the lung function of study patients measured by FEV1 did not change during the study (Table 4). The proportion of patients with mild, moderate, severe, and very severe airflow limitations was generally stable throughout the study. At initial visit, patients with mild airflow limitation accounted for 2.5%, with moderate airflow limitation for 25.1%, with severe for 59.0%, and with very severe for 13.4% (Table 4). Comparable distribution of airflow limitation severity categories can be found in other real-life studies where approximately 10% of patients are categorized with very severe airflow limitation and the patients in the mild category are the least numerous (approximately 2–3%) [18,19,20]. An interesting observation is that the decliners were mainly in the group with milder airflow limitation, which agrees with observations from large clinical trials [21].

Above 75% of patients enrolled in the study experienced exacerbation of COPD, and almost half of them were hospitalized due to exacerbation in the 12-month period preceding the study entry (Figure 2). This proportion is relatively high and shows that COPD leads to a significant medical and financial burden on the Polish healthcare system. Interestingly, 330 patients were defined as ‘decliners’ (the decrease in FEV1 greater than 100 mL was observed during the study conduct), but the rate of exacerbation in this group was similar to that in ‘non-decliners’ (Table 3).

A wide range of exacerbating COPD patient proportions can be found in other real-life studies. In a Bulgarian 1-year prospective observational study, the percentage of patients with exacerbations in the 12-month period before the study was similar, accounting for approximately 75% [22]. Two large observational studies conducted in Central and Eastern European countries, Switzerland and Israel, also demonstrated comparable proportions of exacerbating COPD in similar periods [18,19]. The POPE study, another observational study conducted in Central and Eastern European countries, demonstrated a much lower percentage of exacerbating patients, accounting for 37%) [23]. The German study DACCORD showed that only about 20–25% of COPD patients had exacerbation in the 6-month period prior to the study entry [20,24]. Another German non-interventional study on 3653 COPD patients reported a 35% frequency of exacerbations in the 2-year period preceding the study entry [25].

The categorization of patients by two systems of lung function impairment severity evaluation (GOLD and ATS/ERS) showed that there were no patients categorized simultaneously as GOLD-non-severe and ATS/ERS-severe (FEV1 > 50% pred. and z-score < −4) (Figure 3). Patients categorized by the two systems as non-severe, statistically, were more significantly and frequently decliners (Table 6). Patients classified into different lung function severity groups (non-severe and severe, independent of the method of classification, GOLD or ATS/ERS) had similar exacerbation risk, demonstrating that the new ATS/ERS 2022 strategy for evaluation of lung function impairment severity seems inappropriate for the assessment of exacerbation risk.

The classic risk factors for COPD are male sex and tobacco smoking. According to meta-analysis performed in 2018, the prevalence of COPD among women is 6.16% and among men, 9.23% [26]. In our study, men accounted for 67% of enrolled patients and were also more numerous in ‘decliners’ compared with ‘non-decliners’ (74% vs. 64%, *p* = 0.004). Tobacco smoke is the most common cause of COPD, and smoking cessation is recommended for all smoking COPD patients [27]. In the present study, smokers accounted for 35.8%, whereas ex-smokers for 55.2% (Table 1), which indicates that the vast majority of patients enrolled in our study were exposed to the tobacco smoke in some period of their lives. These results are generally in line with data from other real-life studies showing that at least 75% of studied COPD patients were current or ex-smokers [18,19,20,22,23,24].

In our study, the number of exacerbations and hospitalizations reported by patients at each visit decreased. Other real-life studies have also shown that during the study course, patients usually improve their state, especially in the first 6 months of the study [19,24]. It was observed that shortly after enrolment in the study, patients adhered to the treatment very well and therefore experienced significant health improvement. Other patient-related outcomes, such as severity of dyspnea measured by mMRC score (grade 2 in half of patients) and CAT (median score from 15 to 17 points), were generally stable throughout the study (Figure 2). According to the GOLD 2017–2022 strategy of classification, we identified the following distribution in the study groups A/B/C/D: 6.7%/41.5%/1.4%,/50.4%, respectively. During this study, data on concomitant diseases and medications were collected. Hypertension was the most frequent concomitant disease reported by the study patients, followed by ischemic heart disease, heart failure, diabetes, and hyperlipidemia (Table 1). The high prevalence of cardiovascular diseases in COPD patients has been reported in other real-life studies [18,19,20,25]. Other important comorbidities in COPD are depression and anxiety, which are associated with poor prognosis and the risk of exacerbations [28,29]. It has been demonstrated that the prevalence of depression in COPD patients is approximately 25% [30]. In our study, depression and anxiety disorders were diagnosed in approximately 7% of patients, which may suggest the underdiagnosis of these disorders in the Polish COPD population. The medication taken during the study was stable (Table 2), and there were three combinations of ICS/LABA: extrafine beclomethasone/formoterol, fluticasone propionate/salmeterol, or budesonide/formoterol. Data on the impact of ICS/LABA combination on patient state were not gathered in this study; however, it is known that these three combinations are comparable in terms of their effectiveness and safety, and there is no evidence for the superiority of either ICS/LABA combination in the published literature [31,32,33,34,35,36].

This study has several strengths and limitations. The strong point of this study is its observational, real-world design and long duration, enabling the collection of patient-related outcomes and data describing everyday life of ICS/LABA-treated COPD patients in Poland. This study is the first to analyze the use of ICS/LABA fixed-dose combination in the Polish COPD population and utilized the new ATS/ERS 2022 recommendation for the evaluation of lung function impairment severity [7].

The main limitation of the study was the gradual reduction in participants from visit to visit (from 922 at Visit 1 to 151 at Visit 4), which may have introduced selection bias, as patients who remained under observation could represent a subgroup with higher engagement or treatment stability. The small number of patients at Visit 4 reduces statistical power and limits the generalizability of the findings. However, progressive loss to follow-up is an inherent characteristic of long-term RWE studies and contrasts with RCTs, where retention is actively maintained through structured monitoring protocols [37]. Similarly, not all patients fulfilled the spirometric GOLD criteria for COPD diagnosis; however, the phenomena of overdiagnosis and pharmacological overtreatment in COPD have been repeatedly reported in observational cohorts and real-world clinical practice [16].

Future research should focus on strategies to minimize attrition in RWE designs, such as integrating electronic health record-based follow-up, remote monitoring tools, or patient engagement platforms. Additionally, studies designed to prospectively identify subgroups at higher risk of treatment discontinuation or clinical deterioration could help tailor follow-up intensity and optimize resource allocation within healthcare systems.

## 5. Conclusions

This non-interventional, observational study on COPD patients treated with ICS/LABA in every day medical practice in Poland showed that during the 2-year period, pulmonary function, medical treatment, and patient-related outcomes such as severity of dyspnea measured by mMRC score and CAT were generally stable throughout the study; however, in about 30% of patients, a decrease in lung FEV1 loss greater than 100 mL was observed. The new strategy for evaluating lung function impairment severity according to the new ATS/ERS 2022 recommendations utilizing FEV1 z-score value seems to not add benefit in assessing exacerbation risk. Factors that were found to be associated with the occurrence of exacerbations and were classified into the C/D GOLD category included the presence of comorbidities and increased symptoms. The frequency of exacerbations decreased, especially in the first six months of the study, compared to the initial visit, which is a phenomenon already observed in other non-interventional studies.

## Figures and Tables

**Table 1:** Characteristics of the study patients: demographic data, smoking status, concomitant diseases.

Characteristic	*n* = 965
Age [years]	
Mean (SD)	66.9 (9.4)
Missing	4
Gender, *n* (%)	
Female	311 (32.2)
Male	651 (67.5)
Missing	3 (0.3)
BMI [kg/m^2^]	
Mean (SD)	27.1 (5.4)
Missing	6
Smoker, *n* (%)	
Smoker	345 (35.8)
Ex-Smoker	533 (55.2)
Non-smoker	80 (8.3)
Missing	7 (0.7)
Concomitant diseases; *n* (%)	
Hypertension	568 (70.7)
Ischemic heart disease	249 (31)
Heart failure	165 (20.5)
Diabetes	154 (19.2)
Hyperlipidemia	153 (19.1)
Osteoporosis	60 (7.5)
Depression and anxiety disorder	55 (6.8)
Myopathy (muscle weakness)	45 (5.6)
Persistent atrial fibrillation	36 (4.5)
Paroxysmal atrial fibrillation	33 (4.1)
Lung cancer	7 (0.9)
Other cancers	29 (3.6)
Sleep apneas	15 (1.9)
Other disorders	310 (32.1)

**Table 2:** Pharmacotherapy of patients during the study.

Parameter	Visit 0	Visit 1	Visit 2	Visit 3	Visit 4
Short-acting beta2-adrenergic agonists	416 (43.1%)	328 (37.8%)	275 (38.9%)	214 (39.9%)	70 (46.4%)
LABAs	69 (7.2%)	54 (6.2%)	47 (6.6%)	40 (7.5%)	8 (5.3%)
Short-acting anticholinergics	256 (26.5%)	199 (23%)	161 (22.8%)	122 (22.8%)	45 (29.8%)
LAMAs	600 (62.2%)	511 (58.9%)	481 (68%)	373 (69.6%)	104 (68.9%)
ICS	26 (2.7%)	23 (2.7%)	26 (3.7%)	24 (4.5%)	3 (2%)
ICS/LABA	926 (96%)	813 (93.8%)	704 (99.6%)	525 (97.9%)	147 (97.4%)
Beclomethasone/formoterol	364 (37.7%)	320 (36.9%)	276 (39%)	198 (36.9%)	53 (35.1%)
Budesonide/formoterol	208 (21.6%)	179 (20.6%)	154 (21.8%)	119 (22.2%)	24 (15.9%)
Fluticasone/salmeterol	354 (36.7%)	314 (36.2%)	274 (38.8%)	208 (38.8%)	70 (46.4%)
PD4 inhibitors	2 (0.2%)	2 (0.2%)	2 (0.3%)	1 (0.2%)	0 (0.0%)
Aminophylline	369 (38.2%)	308 (35.5%)	274 (38.8%)	217 (40.5%)	69 (45.7%)
Other drugs	25 (2.6%)	29 (3.0%)	36 (3.7%)	38 (3.9%)	11 (1.1%)

**Table 3:** Odds ratios and 95% confidence intervals for factors associated with exacerbations (univariate analysis and multivariate model).

Variable	Coefficient	Odds Ratio	95% CI	*p*-Value
Male sex	−0.2682	0.7648	0.5631 to 1.0387	0.086
Age (years)	−0.00057465	0.9994	0.9820 to 1.0172	0.9491
BMI kg/m^2^	−0.017148	0.9830	0.9571 to 1.0096	0.2083
Comorbidities	0.44212	1.5560	1.0989 to 2.2033	0.0127
GOLD-severe—ATS/ERS non-severe *	−0.014633	0.9855	0.7152 to 1.3580	0.9287
GOLD-severe—ATS/ERS-severe *	−0.020001	0.9802	0.6148 to 1.5627	0.933
‘Decliner’	−0.069795	0.9326	0.6924 to 1.2561	0.646
mMRC	0.97698	2.6564	2.1632 to 3.2621	<0.0001
Smoking	−0.27197	0.7619	0.5575 to 1.0411	0.0878

* vs. GOLD non-severe ATS/ERS non-severe.

**Table 4:** Airflow limitation grades based on FEV1 expressed as % of predicted value according to GOLD 2017–2022.

Airflow Limitation, *n* (%)	Visit 0	6 Months	12 Months	18 Months	24 Months
Mild (>80%)	20 (2.5)	28 (4.6)	20 (3.7)	15 (4.0)	3 (2.6)
Moderate (50–80%)	198 (25.1)	173 (28.5)	155 (28.9)	117 (31.0)	30 (25.9)
Severe (30–50%)	466 (59.0)	321 (52.9)	293 (54.7)	194 (51.5)	66 (56.9)
Very severe (<30%)	106 (13.4)	85 (14.0)	68 (12.7)	51 (13.5)	17 (14.7)

**Table 5:** Comparison of ‘decliners’ and ‘non-decliners’ populations (baseline data).

Characteristic		Decliners, *n* = 330	Non-Decliners, *n* = 592	*p*-Value
Males, *n* (%)		244 (73.9)	382 (64.5)	0.004 ^a^
Age, years (median, IQR)		66 (60–72)	67 (61–75)	0.026 ^b^
FEV1% pred. median (IQR)		46.62 (38.66–59.22)	42.06 (33.76–53.6)	<0.001 ^b^
FEV1 (z-score) (mean, SD)		−2.96 (1.04)	−3.21 (1)	<0.001 ^c^
FVC % pred. median (IQR)		68.66 (57.41–80.66)	62.92 (52.07–75.3)	<0.001 ^b^
FVC (z-score) mean (SD)		−1.99 (1.17)	−2.29 (1.23)	<0.001 ^c^
GOLD-non-severe/ATS/ERS-non-severe, *n* (%)		139 (42.1)	176 (29.7)	0.004 ^a^
GOLD-severe/ATS/ERS-non-severe, *n* (%)		155 (47.0)	300 (50.7)	
GOLD-severe/ATS/ERS-severe, *n* (%)		36 (10.9)	116 (19.6)	
CAT, median (IQR)		17 (12–22)	17 (11–23)	0.549 ^b^
mMRC grade, *n* (%)	0	1 (0.3)	9 (1.5)	0.004 ^a^
	1	72 (21.8)	105 (17.9)	
	2	176 (53.3)	256 (43.6)	
	3	74 (22.4)	194 (33.0)	
	4	7 (2.1)	23 (3.9)	
Exacerbations, *n* (%)	yes	285 (88.2)	507 (88.6)	0.943 ^a^
	no	38 (11.8)	65 (11.4)	
Ever-smokers, *n* (%)	yes	308 (93.9)	534 (90.5)	0.096 ^a^
	no	20 (6.1)	56 (9.5)	
Cardiovascular disease, *n* (%)	yes	125 (37.9)	234 (39.5)	0.673 ^a^
	no	205 (62.1)	358 (60.5)	

Statistical significance of differences assessed by ^a^—Chi^2^ test; ^b^—Mann–Whitney U test; ^c^—T test.

**Table 6:** Comparison of patients categorized by two systems of lung function impairment severity (GOLD and ATS/ERS), data at baseline.

Characteristic		GOLD-Non-Severe/ ATS/ERS-Non-Severe	GOLD-Severe/ ATS/ERS-Non-Severe	GOLD-Severe/ATS/ERS-Severe	*p*-Value
Males, *n* (%)		190 (60.3)	325 (71.4)	111 (73.0)	0.002 ^a^
Age [years], median (IQR)		67 (60–75)	69 (64–75)	60 (55–64)	<0.001 ^b^
FEV1% pred., median (IQR)		62.68 (55.69–73.6)	40.78 (36.51–45.06)	28.73 (25.26–33.98)	<0.001 ^b^
FEV1 (z-score), mean (SD)		−2.06 (0.88)	−3.43 (0.34)	−4.41 (0.38)	<0.001 ^c^
FVC % pred., median (IQR)		78.16 (67.88–90.72)	60.82 (52.44–71.44)	50.94 (42.93–60.41)	<0.001 ^b^
FVC (z-score), mean (SD)		−1.32 (1.03)	−2.39 (0.99)	−3.36 (0.87)	<0.001 ^c^
Decliners, *n* (%)		139 (44.1)	155 (34.1)	36 (23.7)	<0.001 ^a^
Non-decliners, *n* (%)		176 (55.9)	300 (65.9)	116 (76.3)	
mMRC grade, *n* (%)	0	8 (2.6)	2 (0.4)	0 (0)	<0.001 ^a^
	1	85 (27.2)	68 (15.0)	24 (15.8)	
	2	156 (50.0)	214 (47.2)	62 (40.8)	
	3	60 (19.2)	154 (34.0)	54 (35.5)	
	4	3 (1.0)	15 (3.3)	12 (7.9)	
CAT, median (IQR)		16 (10.25–22)	16 (11–22)	19 (12–24.5)	0.006 ^b^
Exacerbations, *n* (%)	yes	267 (88.4)	394 (88.9)	131 (87.3)	0.866 ^a^
	no	35 (11.6)	49 (11.1)	19 (12.7)	
Ever-smokers, *n* (%)	yes	280 (89.5)	417 (91.9)	145 (96.0)	0.055 ^a^
	no	33 (10.5)	37 (8.1)	6 (4.0)	
Cardiovascular disease, *n* (%)	yes	114 (36.2)	201 (44.2)	44 (28.9)	0.002 ^a^
	no	201 (63.8)	254 (55.8)	108 (71.1)	

Statistical significance of differences assessed by ^a^—Chi^2^ test; ^b^—Kruskal–Wallis test; ^c^—ANOVA.

**Table 7:** Results of MMRC in GOLD categories at baseline.

	GOLD 2017–2022 Category				
mMRC	A	B	C	D	
0	9	1	0	0	10 (1.1%)
1	58	75	15	29	177 (19.3%)
2	0	219	0	213	432 (47.1%)
3	0	80	0	188	268 (29.2%)
4	0	3	0	27	30 (3.3%)
	67 (7.3%)	378 (41.2%)	15 (1.6%)	457 (49.8%)	917

the given percentages are of grand total.

## Data Availability

The original contributions presented in this study are included in the article. Further inquiries can be directed to the corresponding author.
